# Musculoskeletal Diseases and Disorders in the Upper Limbs and Health Work-Related Quality of Life in Spanish Sign Language Interpreters and Guide-Interpreters

**DOI:** 10.3390/ijerph19159038

**Published:** 2022-07-25

**Authors:** Estíbaliz Jiménez-Arberas, Emiliano Díez

**Affiliations:** 1 Facultad Padre Ossó (Centro Adscrito a la Universidad de Oviedo), Calle Prado Picón s/n, 33008 Oviedo, Spain; estibaliz@facultadpadreosso.es; 2 Instituto Universitario de Integración en la Comunidad (INICO), Facultad de Psicología, Universidad de Salamanca, Av. Merced, 109, 37005 Salamanca, Spain

**Keywords:** fatigue, occupational health, quality of work life, sign language, upper extremity

## Abstract

Disorders in the upper limbs are common among sign language interpreters and are related with different risk factors, among which are the difficulties of interpreting work in the educational setting, posture, and emotional together with physical stress. The aim of this study was to inquire about the different musculoskeletal disorders and diseases present in a group of sign language interpreters, and to examine its relationship with the work-related quality of life. A battery of four instruments was administered to 62 sign language interpreters, composed of a sociodemographic data and musculoskeletal disease questionnaire, a health-related quality of life measurement scale (SF-36), a measurement scale of the impact of fatigue (MFIS), and an instrument for assessing hand-function outcomes (MHOQ). All the study participants had presented some kind of musculoskeletal pathology during their work career, such as tendinitis, overuse syndrome, and repetitive strain injury. In addition, many of the participants present difficulties in occupational performance that affect their daily activities. A high percentage, close to 70%, of the interpreters suffer from musculoskeletal disorders, serious enough to modify their activities and affect both the quality of their work as interpreters and their quality of life, with important mediating variables being the number of diseases; physical, cognitive, and social fatigue; and satisfaction with the hand function.

## 1. Introduction

Sign language interpreting services may be considered a powerful solution to the needs of the deaf community, as they aim to break communication barriers and guarantee deaf people’s right to fully participate in community settings in the same conditions as any other individual [1].

For centuries, sign language interpreting was relegated to teachers, social workers, or even relatives. The interpreting profession as such arose in the final decades of the 20th century [2]. Social knowledge and awareness of the deaf community have increased since then. Sign language was officially acknowledged by Spanish Law 27/2007 (October 23rd) [3], which recognises Spanish and Catalan sign languages as official languages in Spain, and promotes regulated training programmes to train sign language interpreters.

Although the sign language interpreters collective (henceforth ‘SLI’) is essential in the context of deaf sign language users, interpreters are often insufficiently acknowledged. Recently, work-related disorders within the SLI collective are awakening some research interest, following the discovery that professional interpreters frequently suffer from musculoskeletal disorders associated with their work performance, although this problem interestingly appears to be less widespread amongst deaf people. 

Upper limb musculoskeletal disorders and diseases are common in the SLI collective, and the main risk factors include the typical difficulties inherent to the position, such as interpreting in educational contexts, posture, and emotional and physical stress, among others [4]. There is a correlation between the requirements and demands of the position and each interpreter’s work style, which increases biomechanical stress on the upper limbs, thus exacerbating musculoskeletal symptoms [5]. These professional demands, in addition to ergonomic and psychosocial stressors, exacerbate and cause an early development of pain, muscle tension, functional limitations, and, ultimately, occupational disability. Previous research has also shown that psychosocial factors such as low self-perceived health status [6] constitute a risk factor for the development of musculoskeletal disorders [7], leading to a high prevalence of mental fatigue.

Research conducted by National Institute for Occupational Safety and Health (NIOSH) found that 92% of SLI suffered some kind of ailment and/or physical discomfort of musculoskeletal origin, although only around 20–30% of the instances were directly linked to their work performance [8]. Feuerstein et al. (1997) [7] conducted a survey among 13,988 SLI, obtaining the following results: participants reported pain, rigidity, burning sensation, numbness or tingling in the neck (74% of participants), the hand or wrist (70%), and the forearm (44%).

Several other studies have also aimed to ascertain the most prevalent musculoskeletal disorders. For example, Podhorodecki and Spielholz (1993) [9] concluded that tendinitis is the most common hand/wrist disorder, and it is indeed more common than nerve diseases or injuries such as carpal tunnel syndrome [10], although the latter is very common among SLI in the educational field [11,12]. Meanwhile, Cohn, Lowry, and Hand (1990) [13] studied overuse syndromes and found that the highest prevalence occurred in tendinitis, synovitis, and entrapment neuropathy. However, other less prevalent musculoskeletal disorders also exist, such as common extensor tendonitis, epicondylitis, rotator cuff tendonitis, and De Quervain syndrome [14].

Regarding improvement factors, Cohn, Lowry, and Hand (1990) [13] concluded that the most appropriate approach is prevention, and therefore it is necessary to limit the length of sessions, modify techniques, and carry out stamina exercises, and provide interpreting students with training to minimise physical stress during their work performance [15]. In general terms, and in relation to treatment, a high percentage of SLI initially require pharmacological and medical treatment [14], followed by massages and other chiropractic services. In summary, evidence suggests that one in four sign language interpreters experience symptoms of musculoskeletal disorders that are severe enough to oblige modification of their daily activities.

The systematic review carried out by Fischer, Marshall, and Woodcock (2012) [16] is of particular interest, as they assessed 23 published articles relating to SLI and musculoskeletal disorders and found that all of them featured either a medium or low level of evidence (5 and 18 studies, respectively). With regard to the factors that cause an impact at the musculoskeletal level, it is worth noting that mechanical exposure, speaker’s rate of speech, and stress factors appear to affect musculoskeletal health. Other factors such as time and length of the presentation, break times, pressure, signing style, background knowledge on the topic, and fear or awareness of the potential injuries can also contribute to musculoskeletal disorders [17]. Many of these pathologies result in repetition work losses, which is detrimental to both the professional interpreter and the deaf people who need the services of an interpreter. 

On the other hand, fatigue, often associated with declining quality of life, is one of the symptoms observed by numerous professionals from diverse fields. Fatigue is characterised by its subjective nature and, much as the in quality of life, depends on multiple biological, behavioural, and psychosocial variables. It is for this reason that it is important to consider physical, cognitive, and psychosocial fatigue.

In summary, the main aim of this paper is to explore the different musculoskeletal disorders and diseases that are common among the SLI collective in Spain, and their relation to work-related quality of life. Concurrently, the specific objectives are: firstly, to assess the health-related quality of life of SLI; secondly, to evaluate upper limb status, specifically the hands, using a specific assessment tool; and finally, to explore the perceived level of physical, cognitive, and psychosocial fatigue.

## 2. Materials and Methods

### 2.1. Participants

The participants were 62 adults (6 men and 56 women) who work as Spanish sign language interpreters and/or interpreters/guides, with an average age of 33.26 years old (SD = 7.58). The sample was selected from various associations for Spanish sign language interpreters; we also contacted the Federation of Spanish Sign Language Interpreters and Interpreter-Guides—FILSE. The following inclusion criteria were used to select the final sample: official Spanish sign language interpreters and/or interpreters/guides; and aged 18 years or over. All participants provided written informed consent before being enrolled in the study; the survey was open for two months. Data provided by participants were treated confidentially in compliance with relevant legislation. The sample for this study was selected using nonprobability convenience sampling. 

See Table 1 for other sociodemographic characteristics of the participants.

### 2.2. Instruments

A total of 12 items comprised the ad hoc survey. The items were classified into three sections: (1) personal data (age, sex, etc.); (2) items about musculoskeletal diseases (diagnosed musculoskeletal diseases, treatment received, etc.); and (3) preventive measures of musculoskeletal diseases. 

#### 2.2.1. SF-36 (The Short Form-36 Health Survey) (Medical Outcomes Study Short Form 36)

The Short Form-36 Health Survey [18] is a health-related quality of life (SF-36) generic measuring tool containing 36 items, which assesses both positive and negative health statuses. We used the Spanish version adapted by Alonso, Prieto, and Antó (1995) [19]. It consists of eight scales representing the health concepts most commonly used in the main health surveys, and the most characteristic aspects of the disease and its treatment. The 36 items included in the tool cover the following scales: physical functioning, physical role, body pain, general health status, vitality, social functioning, emotional role, and mental health. Each scale is scored between 0 (worst perceived health status) and 100 (best perceived health status).

#### 2.2.2. MHOQ (Michigan Hand Outcome Questionnaire)

This [20] assessment tool evaluates the outcomes for subjects with hand/wrist pathologies or injuries. We used the Spanish version adapted by Miranda et al. (2008) [21]. Following its preclinical development, a 37-item questionnaire was developed. Each item has a response range from 1 to 5, and the total of the items contained in one domain represents a score between 0 and 100 and is organised into six subscales: (1) general hand function, (2) activities of daily living, (3) pain, (4) work performance, (5) aesthetics, and (6) patient satisfaction regarding hand function. Except for pain and work performance subscales, which are accounted for both sides, the other subscales should be administered separately for left and right hands, in which the total number reaches up to 57 questions. Every subscale receives a score from 0 to 100, with zero being the worst and 100 for the best ever result except for pain. 

#### 2.2.3. MFIS (Modified Fatigue Impact Scale)

MFIS [22] is a multidimensional scale intended to assess the impact of fatigue on the subject’s cognitive, physical, and psychosocial function. We used the Spanish version adapted by Kos et al. (2005) [23]. The MFIS is recommended for both research and clinical practice purposes. It has also been demonstrated that there are no differences in the administration of MFIS across various cultures and languages, including Spanish. The scale consists of 21 items arranged into three subscales: physical, cognitive, and psychosocial. The final score ranges from 0 to 84, with 38 representing the cutoff point in defining the presence of fatigue or nonfatigue. Although this tool was originally intended for the multiple sclerosis population, it has also been used on other populations including those suffering from Parkinson’s disease [24,25] or cerebrovascular accident [26].

### 2.3. Procedure

Data were collected using a custom-made sociodemographic questionnaire and the SF-36 [18,19], MHOQ [20,21], and MFIS [22,23] scales, all of which were available in an online form.

The inclusion criteria were that the participants were qualified technicians in sign language interpretation and guide-interpretation, and that they were active as sign language interpreters (regardless of the type of working day). The exclusion criteria were that they were active, although worked with deaf people but not with job functions and skills as a sign language interpreter or guide-interpreter, and students in advanced cycle internships in sign language interpretation. The study was carried out for a year. Firstly, the Spanish Federation of Sign Language Interpreters and Interpreter-Guides (FILSE) was contacted as the main representative of sign language interpreters, the first contact was made via email when the objectives of the study were described and the link that gave access to the study was attached. The questionnaire’s first question was informed consent according to Bioethic Committee and Law 3/2018, of December 5, on the Protection of Personal Data and Guarantee of Digital Rights, which was requested in this email that it be sent from FILSE to the different associations of the different autonomous communities. The questionnaire was open for 3 months. After its closure, 7 people were excluded for having the training but not working as sign language interpreters, although instead working as sociohealth professionals, mostly with this training as a complement (psychologists, social workers, speech therapists, etc.).

Sixty-two participants from FILSE for SLI were invited to complete the various tools, and the survey was completed by a total of 62 participants. The study design was nonexperimental, cross-sectional, and correlational. All procedures performed in studies involving human participants were in accordance with the ethical standards of the institutional and/or national research committee, and with the 1964 Helsinki Declaration and its later amendments or comparable ethical standards and approved by the Bioethics Committee of the Principality of Asturias (number 2020.244). In the questionnaire provided to the participants, the first question was in relation to consenting to the publication of the data anonymously, following the current Organic Law 3/2018, of December 5, on the Protection of Personal Data and Guarantee of Digital Rights.

## 3. Results

### Data Analysis

We first describe the results related to the musculoskeletal disorders reported by the participants. It is worth noting that all the participants experienced at least one musculoskeletal disorder throughout their professional career and indicated that these diseases had affected their quality of life and perceived health status. Table 2 presents the frequency of the disorders noted by the participants. Treatments received for such diseases can be seen in Table 3.

Table 4 shows the average scores in each dimension of the SF-36 for perceived health status. As some variables were not normally distributed, winsorized mean (level = 0.2) is also provided. The highest scores are obtained in the dimensions of physical function and physical function limitations, while the lowest scores refer to the dimensions of vitality, energy or fatigue, mental health, and emotional function limitation.

Table 5 represents the scores obtained in the MHOQ subscales, which displayed greater satisfaction in the subscales related to activities of daily living and hand-function satisfaction, and lower scores in the aesthetics and pain subscales. The table also shows the scores from the MFIS total scale and its three subscales: physical fatigue, cognitive fatigue, and psychosocial fatigue. 

The SF-36 results allow us to assert that the interpreters in the sample believe that both their mental and general health perception has been affected, and that there is a high level of physical and mental stress among these professionals, as we can infer from the MFIS and SF-36 scores. Although the global average MFIS score was 28.39 (18.94), which does not exceed the 38-point threshold necessary to confirm a fatigue condition, 16 participants (23.9% of the total sample group) did display global scores above 38.

The results derived from the MHOQ scale revealed that many participants struggle to perform some activities of daily living, particularly certain items with negative scores which the participants considered difficult or very difficult, such as: washing the dishes, opening a jar, lifting a pot, or functioning in their work environment (difficulty in performing the job due to hand/wrist pain, less effective work time, and hand pain). Moreover, several participants face work limitations due to the state of their upper limb.

**Figure 1:**
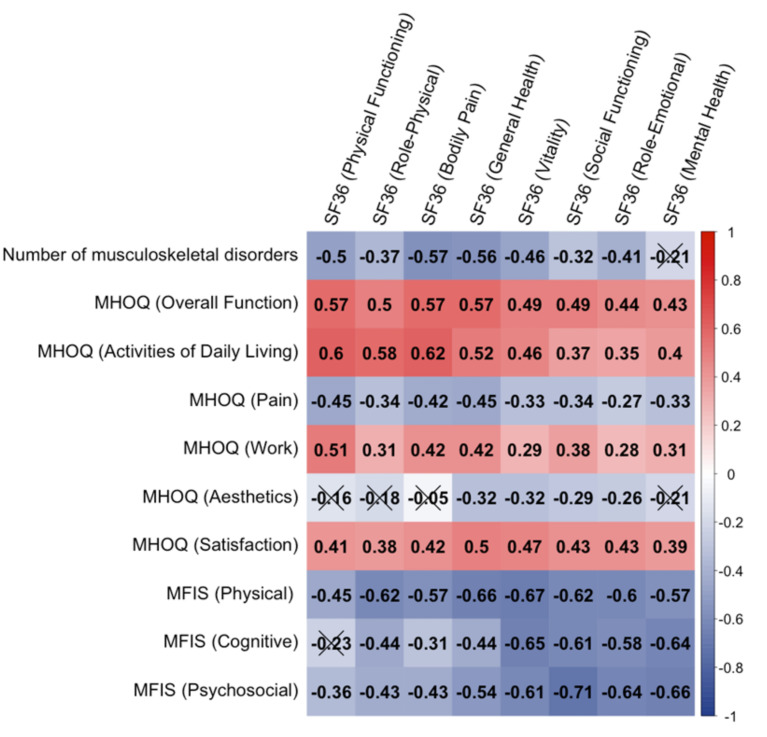
Correlogram representing the Spearman correlation coefficient regarding the number of musculoskeletal disorders, various dimensions related to the hand function (MHQ) and fatigue (MFIS), and the 8 quality of life dimensions of the SF-36 scale. The colour and brightness of each square represent the magnitude and sign of each correlation (red = positive, blue = negative), respectively. The X indicates a nonsignificant correlation (*p* > 0.05; with Bonferroni–Holm correction).

Finally, we carried out an analysis to explore the relationship between the number of musculoskeletal disorders, fatigue (MFIS), hand function (MHOQ), and quality of life (SF-36). Results are shown in Figure 1.

As it can be seen in Figure 1, most correlations were significant and, in some cases, showed medium–high values. On the one hand, we can see a consistent relationship between the number of musculoskeletal disorders noted by the participants and the various dimensions of quality of life, particularly pain and general health. In terms of hand-function satisfaction, most MHOQ dimensions also showed significant correlations with most SF-36 dimensions (with the exception of hand aesthetic appearance satisfaction). A major negative relationship pattern was observed between physical, cognitive and psychosocial fatigue, and quality of life.

A set of hierarchical regression analyses were conducted to supplement and explore the pattern of correlations in greater depth. Specifically, for each dimension of the SF-36, the predictive value of the variables related to fatigue (MFIS), hand satisfaction (MHQ), and number of neuromuscular diseases (N-ENM) was tested with three successive multiple regression models that incrementally included the previous model variables. Table 6 shows the increment of explained variance for each step and also the final explained variance.

The regression analysis showed that fatigue variables (MFIS) best explained the quality of life variance in all dimensions of the SF-36, particularly those assessing social function (57% variance) and mental health (51% variance). Hand satisfaction (MHOQ) was relevant only to physical function (21% variance). The number of diseases could be a relevant factor to the general health (6% variance), vitality (4% variance), and emotional role (6% variance) dimensions, although its explanatory value was low.

## 4. Discussion

Firstly, the present study demonstrates a high incidence of musculoskeletal disorders among Spanish SLI, which is also related to a decrease in health-related quality of life. According to the European Agency for Safety and Health at Work, about three in five workers in the European Union report complaints of musculoskeletal conditions, predominantly back pain and muscle aches in the upper extremities [27]. It also found that of all workers in the European Union with a work-related health problem, 60% identified musculoskeletal problems as a serious problem. In this same report, working women were reported to have a higher prevalence of these diseases. In this study, 90.3% participants were women compared to 9.7% men. Although musculoskeletal disorders are a factor that increases significantly with age, in this study we found a mean of 33.26 years, a noteworthy result derived from the age of the participants [27]. Additionally, in the report of the European Agency for Safety and Health, they indicated that physical risk factors such as posture or repetitive work [27] and both factors are present in SLI, adding that 21 related organizational and psychosocial risk factors were reported, with at least one of the three types of diseases studied: back pain and upper and lower limb musculoskeletal diseases. However, this report indicates the need for further testing to confirm specific risk factors, together with psychosocial factors. In Spain, there is a great dearth of scientific literature regarding these risk factors and prevalence of musculoskeletal diseases and other risk factors such as fatigue in SLI [28]. 

SF-36 had previously been used in other Spanish studies to assess the overburden level and the impact of work on the quality of life, although no Spanish empirical studies address this matter in relation to the interpreting field [15].

Several studies have addressed musculoskeletal diseases in this group, or even specific pathologies such as work-related shoulder pain, although their results are not generalizable as they found a high prevalence [8]. Moreover, this relationship may be affected by variables such as hand function and physical, cognitive, and psychosocial fatigue. The results of this study are consistent with this idea since a pattern of moderate negative correlations between quality of life and physical, cognitive, and psychosocial fatigue was observed. Additionally, regression analysis showed that fatigue variables exhibited the largest increases in explained variance of quality of life. The results also showed that the different dimensions of quality of life, especially physical pain, general health, and physical functioning, are positively related with global hand function, although it is the physical functioning that seems to be best explained by variables related to hand functioning.

Feuerstein and Fitzgerald [5] demonstrated an interaction between work demands and upper extremity cumulative trauma disorders. As in other studies, tendinitis (*n* = 30) [9,13] and tension neck syndrome (*n* = 31) [7] display the highest prevalence, together with anti-inflammatory medication (*n* = 28) and other drugs such as analgesics in terms of treatment [7]. Secondly, this study also demonstrates the high incidence of other pathologies such as tendinitis (*n* = 30) [9,13] and tension neck syndrome (*n* = 31) [7,29]. We must keep in mind that musculoskeletal disorders are 1 of 10 diagnostic groups that increased in size by more than 30% from 1990 to 2010 [30] within the general population and that they result in serious hand injury, chronic pain, and fatigue correlated with low quality of life, anxiety, mental fatigue, and depression [31]. 

However, most of the treatments received are drugs and manual physical therapy, yet no participant reported receiving prevention or articular economy education to prevent or relieve these disorders. 

Upper limb diseases represent a considerable economic burden and productivity loss [32]. These pathologies can lead to SLI not being productive in their daily work and this leads to a limitation in communication between deaf and hearing people; in addition, the diagnosis of these musculoskeletal diseases can lead to sick leave by these professionals. The results of the present study, although not extrapolated, do seem to indicate an affectation in the mental health of these professionals, since work in the social and health sector is stressful and is already associated with the risk of depression, anxiety, burnout, and mood and sleep disorders [33]. Together with the professionals’ physical health, these can lead to a lower quality of working life. In this same line, the scientific literature has addressed that generally women present worse mental health conditions and general health issues than men [34]; it should be highlighted that in the present study 90.3% of the participants were women [35]. More than two decades ago, Scheuerle [15] reported the importance of preventing these pathologies, reducing interpretation times, and modifying the techniques themselves. The work of the SLI is a job with great physical and psychological exhaustion, where attention and working memory are essential, so not only physical but cognitive and psychosocial fatigue can lead to stress, among other mental health issues. The difficulties in the work and occupational performance of these professionals, in relation to the social and attitudinal environment in which they develop, together with the personal factors that may predispose to the development of these diseases, make it necessary to prevent these pathologies in order to minimize their consequences, including the personal and social effects. Hence, the results of the study point to future lines of research, such as prevention studies or programmes, as they are already being developed in other countries.

Finally, we would like to acknowledge that one of the most prominent limitations of the present study relates to the sampling method (i.e., convenience non-probabilistic sampling) and the sample size (small and heterogenous). This fact clearly limits the generalisation of the results to the collective as a whole. Although the sample is small, the Spanish context must be understood. There are no recent data on the number of active sign language interpreters, but according to the State Confederation of Deaf People (CNSE), there are 2781 accredited interpreters, although only 25% have worked or actively work in this profession [36]. The professionalization process must be taken into consideration, since the first official service of mimic interpreters was not established until 1987, and in 1990 the first association of sign language interpreters was established. Further, it was not until 1998 that the Higher-Level Vocational Training Cycle of Technician in Sign Language Interpretation began to be taught. Lastly, in the 2016–2017 academic year, the Degree in Spanish Sign Language and the Deaf Community was first established, with two promotions for these graduates. Limitations are also present in terms of the measuring tools employed, which primarily assess subjective perceptions among participants. It is also worth noting that we did not adopt a longitudinal perspective to explore the impact of musculoskeletal disorders on occupational performance. 

It will therefore be necessary to expand this line of research and to conduct thorough studies employing both quantitative and qualitative methodologies, which will allow us to understand the observed results in greater depth. It would equally be of interest to carry out specific training and prevention programmes in order to minimise the incidence of musculoskeletal diseases and their general and mental health consequences. It is necessary for other organizations, for example, the European Survey on Working Conditions (EWCS), to include perception of fatigue and quality of life as a parameter for musculoskeletal diseasein SLI. Considering not only physical but also cognitive and psychosocial aspects is important for developing better social, employment, and work policies.

## 5. Conclusions

In summary, we may conclude that Spanish sign language interpreters and guide-interpreters that participated in this study reported suffering from musculoskeletal disorders with a high prevalence of pathologies, such as tension neck syndrome and tendinitis, present in half of the participants, together with other high-frequency pathologies such as repetitive strain injury and overuse syndrome. Furthermore, the scores obtained in their general health, more specifically mental health, fatigue, and hand function, have an impact on their health work-related quality of life. All these factors can limit their professional practice, and also could have an impact on deaf people who request interpretation services.

We also acknowledge the imperative need to continue research in this specific field as a way to obtain evidence allowing us to define musculoskeletal disorders as typical work-related illnesses within the Spanish SLI collective, to improve their health work-related quality of life, but also for the deaf people who need their services, since these professionals are often the crucial link between the deaf and hearing communities. 

## Figures and Tables

**Table 1:** Sociodemographic profile of sign language interpreters (SLI) and interpreters/guides and musculoskeletal disorders (*n* = 62).

Variable	N (%)
Age	M = 33.26; *SD* = 7.520
Sex	
Male	6 (9.7)
Female	56 (90.3)
Employment status	
Active	53 (85.5)
Inactive	9 (14.5)
Occupation	
SLI	45 (72.6)
SLI and interpreter/guide	8 (12.9)
SLI and social worker	2 (3.2)
SLI and educator	1 (1.6)
SLI and teacher	1 (1.6)
SLI and professor	1 (1.6)
SLI/guide	1 (1.6)
SLI trainer	3 (4.8)
Work experience	
<1	25 (37.3)
1–2	2 (3)
3–5	11 (16.4)
5–10	9 (13.4)
10–15	9 (13.4)
15–20	3 (4.5)
>20	3 (4.5)

**Table 2:** Frequency of musculoskeletal disorders and diseases in sign language interpreting and interpreting/guiding (*n* = 62).

Musculoskeletal Disorders	Frequency/%
Tension Neck Syndrome	31
Tendinitis	30
Overuse Syndrome	20
Repetitive Strain Injury	19
Medial Epicondylitis	8
Digital Neuropathy	7
Rotator Cuff Tendonitis	6
Thoracic Outlet Syndrome	5
Trauma	5
Carpal Tunnel Syndrome	4
Tenosynovitis of hand and wrist extensors	4
Wrist Bursitis	4
Pronator Teres Syndrome	4
Stenosing Tendosynovitis	4
Arthritis	3
Tenosynovitis	3
Arthrosis	2
Peritendinitis	2
Subacromial Syndrome	1
Epicondylitis	1
Nerve Entrapment Syndrome	1
Spine deviations	1

**Table 3:** Upper limb treatments received by participants (*n* = 62).

Treatment	Frequency
Massage	45
Heat	38
Anti-inflammatory	28
Analgesic	22
Rest	22
Ultrasound	13
Cold	13
Splint	12
Bandage	8
Paraffin	4
Taping	3
Laser	2
Surgical	2

**Table 4:** Scores obtained in SF-36 (*n* = 62).

SF-36 Dimension	M (SD)	Winsorized Mean
General Health Scale (GH)	66.44 (18.05)	67.54
Physical Functioning Scale (PF)	93.63 (10.1)	95.00
Role—Physical Scale (RP)	78.88 (36.21)	86.64
Bodily Pain Scale (BP):	69.19 (20)	71.01
Social Functioning Scale (SF)	76.01 (22.44)	77.42
Mental Health Scale (MH)	65.68 (20.5)	65.87
Role—Emotional Scale (RE)	67.74 (43.51)	67.74
Vitality Scale (VT)	54.92 (20.95)	55.42

**Table 5:** Scores obtained in the six subscales of the MHOQ (*Michigan Hand Outcome Questionnaire*) and scores obtained in MFIS (*Modified Fatigue Impact Scale*) (*n* = 62).

Subscale MHOQ	M (SD)	Winsorized Mean
MHOQ (Overall Function Subscale)	70.80 (23.6)	72.02
MHOQ (Activities of Daily Living Subscale)	92.5 (13.23)	95.04
MHOQ (Work Performance Subscale)	83.7 (20.49)	87.58
MHOQ (Aesthetics Subscale)	15.32 (20.42)	11.69
MHOQ (Patient’s Hand-Function Satisfaction Subscale)	71.50 (24.16)	74.26
MHOQ (Pain Subscale)	21.37 (19.12)	19.27
MFIS (Physical Fatigue)	12.48 (8.1) (0–36 score)	11.94
MFIS (Cognitive Fatigue)	13.35 (9.57) (0–40 score)	12.55
MFIS (Psychosocial Fatigue)	2.55 (2.12) (0–8 score)	2.21
MFIS (total)	28.39 (18.94) (0–84 score)	26.7

**Table 6:** Increment of explained variance in the dimensions of the SF36 scale as a function of including fatigue (MFIS), hand satisfaction (MHOQ), and number of neuromuscular diseases (N-ENM) variables in successive models. Adjusted *R*^2^ shows total variance explained by all the predictors in the last step.

	Δ*R*^2^			Adjusted *R*^2^
	Model 1 MFIS	Model 2 MFIS + MHOQ	Model 3 MFIS + MHOQ + N-ENM	
SF36 (Physical Functioning)	0.28 ***	0.21 **	0.02	0.42
SF36 (Physical Role)	0.48 ***	0.06	0.003	0.44
SF36 (Physical Pain)	0.42 ***	0.12	0.03	0.47
SF36 (General Health Status)	0.43 ***	0.04	0.06 *	0.43
SF36 (Vitality)	0.48 ***	0.05	0.04 *	0.49
SF36 (Social Function)	0.57 ***	0.03	0.02	0.53
SF36 (Emotional Role)	0.44 ***	0.05	0.06 **	0.46
SF36 (Mental Health)	0.51 ***	0.04	0.002	0.47

*** *p* < 0.001, ** *p* < 0.01, * *p* < 0.05.

## Data Availability

The raw data supporting the conclusions of this article will be made available by the authors, upon reasonable request.
